# The Generation of a Lung Cancer Health Factor Distribution Using Patient Graphs Constructed From Electronic Medical Records: Retrospective Study

**DOI:** 10.2196/40361

**Published:** 2022-11-25

**Authors:** Anjun Chen, Ran Huang, Erman Wu, Ruobing Han, Jian Wen, Qinghua Li, Zhiyong Zhang, Bairong Shen

**Affiliations:** 1 Institutes for System Genetics West China Hospital Chengdu China; 2 iHealthd Shanghai Inc Shanghai China; 3 Guilin Medical University Affiliateted Hospital Guilin China; 4 Guilin Medical University Guilin China

**Keywords:** lung cancer, risk factor, patient graph, UMLS knowledge graph, Unified Medical Language System, connection delta ratio, EMR, electronic health record, EHR, electronic health record, cancer

## Abstract

**Background:**

Electronic medical records (EMRs) of patients with lung cancer (LC) capture a variety of health factors. Understanding the distribution of these factors will help identify key factors for risk prediction in preventive screening for LC.

**Objective:**

We aimed to generate an integrated biomedical graph from EMR data and Unified Medical Language System (UMLS) ontology for LC, and to generate an LC health factor distribution from a hospital EMR of approximately 1 million patients.

**Methods:**

The data were collected from 2 sets of 1397 patients with and those without LC. A patient-centered health factor graph was plotted with 108,000 standardized data, and a graph database was generated to integrate the graphs of patient health factors and the UMLS ontology. With the patient graph, we calculated the connection delta ratio (CDR) for each of the health factors to measure the relative strength of the factor’s relationship to LC.

**Results:**

The patient graph had 93,000 relations between the 2794 patient nodes and 650 factor nodes. An LC graph with 187 related biomedical concepts and 188 horizontal biomedical relations was plotted and linked to the patient graph. Searching the integrated biomedical graph with any number or category of health factors resulted in graphical representations of relationships between patients and factors, while searches using any patient presented the patient’s health factors from the EMR and the LC knowledge graph (KG) from the UMLS in the same graph. Sorting the health factors by CDR in descending order generated a distribution of health factors for LC. The top 70 CDR-ranked factors of disease, symptom, medical history, observation, and laboratory test categories were verified to be concordant with those found in the literature.

**Conclusions:**

By collecting standardized data of thousands of patients with and those without LC from the EMR, it was possible to generate a hospital-wide patient-centered health factor graph for graph search and presentation. The patient graph could be integrated with the UMLS KG for LC and thus enable hospitals to bring continuously updated international standard biomedical KGs from the UMLS for clinical use in hospitals. CDR analysis of the graph of patients with LC generated a CDR-sorted distribution of health factors, in which the top CDR-ranked health factors were concordant with the literature. The resulting distribution of LC health factors can be used to help personalize risk evaluation and preventive screening recommendations.

## Introduction

Early lung cancer (LC) detection is a key strategy to combat this deadly disease worldwide [1]. The National Lung Screening Trial in the United States and similar clinical trials around the world have shown an approximately 20% reduction in mortality from LC as a result of screening with low-dose computed tomography [2]. Based on these studies, LC screening medical guidelines as well as statistical risk prediction models including PLCO_M2012_ have been implemented to recommend screening for smokers [3]. However, screening is not commonly recommended for nonsmokers even though they represent a significant percentage of patients with LC worldwide, 15%-20% among male patients and over 50% among female patients [4]. In addition, adoption of LC screening is still very low. For example, only approximately 5% of the at-risk population received their annual screening in the United States [5].

Risk-based or personalized screening approaches are being studied to overcome these challenges [6]. We believe that a deeper understanding of the spectrum of risk factors for LC and applying technologies such as machine learning and knowledge graphs (KGs) will generate more cost-effective screening solutions.

KGs have been widely applied in biomedical research. For interpreting proteomics data, a large-scale clinical KG has been plotted from biomedical data using the Neo4j tool [7]. Open-source graph databases and tools including Neo4j have made it easier to build and analyze KGs [8]. Studies have also demonstrated that construction of high-quality patient KGs from electronic medical records (EMRs) using rudimentary concept extraction is feasible and that the KGs can be used to predict diagnosis on the basis of symptoms [9]. Even though graphical representation of patient data holds the promise to illuminate insights in health care and to transform such insights gleaned from EMR data into actionable knowledge, the application of EMR-wide graphs for studying individual disease diagnosis journeys or treatment processes is still limited [10]. A graphical data model has been constructed, integrating clinical and molecular data of patients with non–small cell LC in the Cancer Genome Atlas LC data sets [11]. Another recent study of synthetic patients proposed a new graphical method to identify any particular disease’s potential risk factor distribution from EMR (personal communication by A Chen, March 1, 2022).

The Unified Medical Language System (UMLS) ontology, freely available from the National Library of Medicine, is a KG consisting of millions of nodes and relationships [12]. It forms the foundation of interoperable biomedical information systems and services, including electronic health records. Connecting the UMLS KG to patient graphs may enable semantic search of patient data and support clinical decision-making [13].

This study aimed to construct a patient health factor graph for LC from a hospital EMR and integrate it with the UMLS KG for graph search and risk factor analysis. Through graph search, the study also aimed to generate a distribution of LC health factors, which was expected to help implement personalized LC risk evaluation for preventive screening.

## Methods

### EMR Health Factor Data Collection

We deidentified the patient records from January 2018 to June 2021 and saved them on a secured data server controlled by the hospital’s informatics department. The data set had approximately 1 million patients and 7 million encounters including both outpatients and inpatients, in which patient names, dates of birth, contacts, and addresses were removed. The original identifiers of patients and encounters were replaced by irrelevant random numbers. Before using the data, our research team members were trained in the hospital’s patient data security and privacy policy.

Because the EMR data had no usable codes associated with the diagnoses, synonyms of LC in Chinese were used to search for patients with LC. A total of 1397 patients with LC aged ≥30 years were included in the target data set. The same number (n=1397) of patients without LC and aged ≥30 years were randomly selected as control (or background) patients for comparison purposes.

Deidentified records of outpatient and inpatient visits, diagnoses, laboratory tests, and procedures were imported into a custom data collection tool on the secured data server. The data tool automatically extracted laboratory test data and saved them in the database. Researchers manually selected data from text records and entered them into the database. Because the records were not coded, practical rules were developed to improve consistency in the data collection process. Synonyms were automatically converted to “local standard terms” and the resulting data were called “local standard data.” For each patient, only data from before the final diagnosis of LC were collected for studying disease risk factors, and a patient diagnosis journey (PDJ) object was created in the data tool to contain 1 or multiple encounters leading to the final diagnosis. When exporting PDJ data to a CSV file for analysis, only the latest data for each health factor in PDJ were selected. The final raw data set contained near 50,000 data from patients with LC and over 58,000 data from background patients. There were over 3000 different health factors identified in these data.

### Patient Graph Construction

To simplify the patient graph, continuous numerical data were converted to categorical data. For example, values of age were converted to categories (ranges), including 30-50, 50-70, and >70 years; the value of drinking was “true” if the patient consumed >1 drink per day; the value of smoking was “true” if the patient smoked >1 cigarette per day. Laboratory findings from the EMR were already recorded as categorical variables: normal or abnormal; true or false; positive or negative; high, medium, or low; and up, down, or normal. After value conversion, approximately 93,000 standard data for about 550 factors (ie, codes) that appeared in at least 10 patients with LC were selected and saved into a factor import CSV file. The format of the factor import file was as follows: virtual-id, category, code, term, value, unit, converted-value, and date. Patients with LC and background patients (N=2794) were both saved in a patient import file, one patient per line, with the following format: virtual-id, LC-label (1 for LC, 0 for background), and factor-count.

We used the Neo4j Desktop tool (version 4.4) available freely from Neo4j Inc, which is a graph database with a graphical user interface (Neo4j Browser) to query with Cypher language and view graphs. It provides an application programming interface through a Python driver. It can load data from CSV files to construct graphs. In our patient-centered graph model, each patient was represented by a “Patient” node (total of 2794 patient nodes), while health factor and value pairs were represented by 650 factor nodes. Because all values were categorical and some health factors had more than 1 piece of categorical data, the number of factor-value pair nodes increased from 550 to 650. The health factors were further subdivided into the following categories: Condition, Symptom, Observation, History, RiskFactor, Labtest, Procedure, Medication, and Treatment. The graph drew over 93,000 connections from patients to factors. Constraints were created on each label to ensure uniqueness. Patient nodes required virtual-id while all factor nodes required category, code, and converted-value as node key.

### UMLS Disease Subgraph Construction

**Figure 1:**
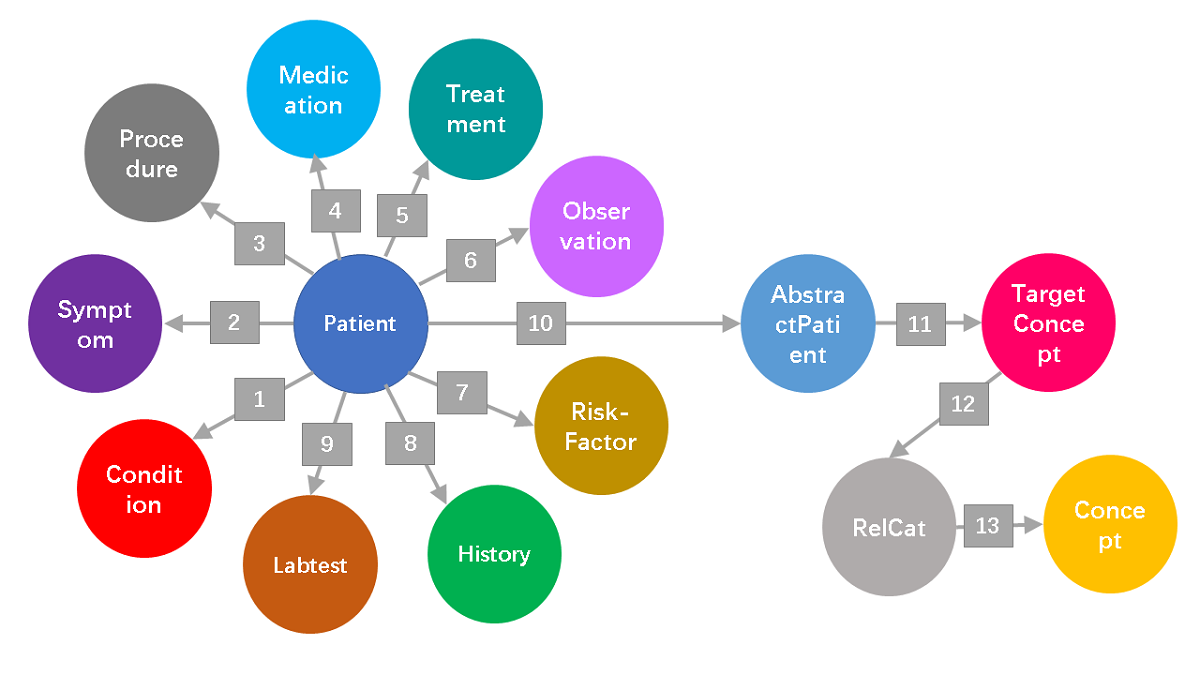
Biomedical graph model for the integration of the electronic medical record patient graph with the Unified Medical Language System knowledge graph of lung cancer. Numbered relationship labels are listed in Table 2.

The UMLS 2020AB release was downloaded from the National Library of Medicine’s UMLS website and installed locally by following the provided instructions. The local UMLS ontology had 2.8 million concepts, 8.3 million terms, and 39.1 million relationships. For generating an LC UMLS subgraph, we directly used the concept file MRCONSO.RRF and relation file MRREL.RRF in rich release format to generate Neo4j graph import files. The LC codes were first expanded to a more complete set of LC codes using the UMLS hierarchy (Table 1). We then used the expanded concept unique identifiers to find all horizontal relations (approximately 1100) between these LC target concepts and other biomedical concepts from over 39 million relations in UMLS ontology. The relations discovered were filtered by a selected set of UMLS relationship attributes for biological or medical concepts (Textbox 1); these were categorized into either biological concept relationships (called “biorel”) or medical concept relationships (called “medrel”). To visualize this simple categorization of biomedical knowledge, we added RelCat nodes between TargetConcept nodes and related Concept nodes in the UMLS subgraph as shown in Figure 1. We then introduced a single AbstractPatient node to connect with all LC TargetConcept nodes. Connecting the patient nodes in EMR graph to the single AbstractPatient node resulted in an integrated biomedical graph that can present any patient’s health factors together with biomedical knowledge from UMLS ontology for LC.

**Table 1:** Expanded lung cancer concepts in the Unified Medical Language System (UMLS) hierarchy.

UMLS concept unique identifiers	Term	SNOMEDCT code
C0581834	Suspected lung cancer	162573006
C0242379	Malignant neoplasm of lung	363358000
C0149925	Small cell carcinoma of lung	254632001
C0007131	Non-Small Cell Lung Carcinoma	254637007
C0152013	Adenocarcinoma of lung (disorder)	254626006
C0149782	Squamous cell carcinoma of lung	254634000
C1306460	Primary malignant neoplasm of lung	93880001
C0153676	Secondary malignant neoplasm of lung	94391008

**List of Unified Medical Language System (UMLS) relationship attributes and categories.**

**Biological concept relationships:**

- gene_associated_with_disease
- gene_involved_in_pathogenesis_of_disease
- gene_mapped_to_disease
- gene_product_malfunction_associated_with_disease
- gene_product_is_biomarker_of
- may_be_cytogenetic_abnormality_of_disease
- may_be_molecular_abnormality_of_disease

**Medical concept relationships:**

- may_treat
- regimen_has_accepted_use_for_disease
- has_associated_finding
- associated_finding_of
- associated_disease
- is_finding_of_disease
- related_to
- clinically_associated_with
- co-occurs_with
- may_be_associated_disease_of_disease
- may_be_finding_of_disease

**Table 2:** Node and relationship labels in the integrated biomedical graph model (shown in Figure 1).

Number	From node label	Relationship labels	To node label
1	Patient	HAS_CONDITION	Condition
2	Patient	HAS_SYMPTOM	Symptom
3	Patient	HAS_PROCEDURE	Procedure
4	Patient	HAS_MEDICATION	Medication
5	Patient	HAS_TREATMENT	Treatment
6	Patient	HAS_OBSERVATION	Observation
7	Patient	HAS_RISKFACTOR	RiskFactor
8	Patient	HAS_HISTORY	History
9	Patient	HAS_LABTEST	Labtest
10	Patient	INSTANCE_OF	AbstractPatient
11	AbstractPatient	MAY_HAVE_TARGET	TargetConcept
12	TargetConcept	HAS_RELCAT	RelCat
13	RelCat	HAS_RELA	Concept

### Patient Health Factor Distribution

We developed a Python script to automatically query the patient graph with each of the health factors. The number of connections from each factor to LC target patients (depicted as “TPC” in equation 1) and background patients (depicted as “BPC” in equation 1) in the search results were counted separately. For each factor, the delta of patient connection counts was calculated by subtracting the number of background patient connections from that of the target patient connections. Division of the delta by the total number of patient connections yielded the “connection delta ratio” (CDR), a relative measure of the strength of connections from a factor to the target patient. Sorting factors by CDR and plotting a graph of CDR versus the sorted factors yielded a distribution of LC health factors from high to low strength.

CDR = (TPC – BPC) / (TPC + BPC) **(1)**

A CDR between 1 and 0 implied that the factor was more related to the target patient, 1 being most related. A CDR below 0 implied that the factor was more related to the background patient.

In this study, factors with a CDR of >0.5 and having connections with at least 10 patients with LC were selected for literature verification. The local standard terms were first translated to English and the corresponding UMLS concepts as well as standard codes from SNOMEDCT_US, LOINC, or RxNORM if possible. We then searched the research literature on Google, Google Scholar, PubMed for each health factor and reviewed the published studies to verify whether the health factors were confirmed risk factors, correlated with LC, were unrelated to LC, or had an unsure relationship with LC. If a factor’s relationship with LC was inconclusive in existing research reports, the factor was tagged as “unsure.” For example, to look up the factor “Hypocalcemia,” search terms included “Lung cancer risk factor Hypocalcemia” and other variations if necessary.

### Ethical Considerations

This retrospective study of EMR patient data has been approved by the institutional review board of Guilin Medical University Associated Hospital in China (QTLL202139).

## Results

### Integrated Graph Model of the EMR Patient Graph and the UMLS KG

To study the spectrum of health factors related to LC in the hospital EMR, we applied a new graph method that we recently developed using synthetic patient data. Figure 1 shows the graph model integrating the EMR patient graph and UMLS knowledge subgraph for LC. The patient graph is patient-centered with patient nodes connecting to different categories of health factor nodes. Table 2 lists the relationships between nodes, as generated in the graph database. The UMLS subgraph in this model is focused on the horizontal biomedical relationships between LC nodes and related concept nodes. Such an integration model enables the presentation of a patient’s actual health factors together with the UMLS KG’s related biomedical factors in the same graph.

### Patient Health Factor Graph Based on EMR Data

From the hospital EMR, 1397 patients with LC were selected along with the same number of background patients without LC. After deidentified data of laboratory tests and procedures were integrated into the corresponding encounters, a total of 108,000 standard data for various categories of health factors were extracted from patient encounters. Although over 3000 different factors were collected, only approximately 550 factors shared by at least 10 patients with LC were used for building the patient health factor graph.

**Figure 2:**
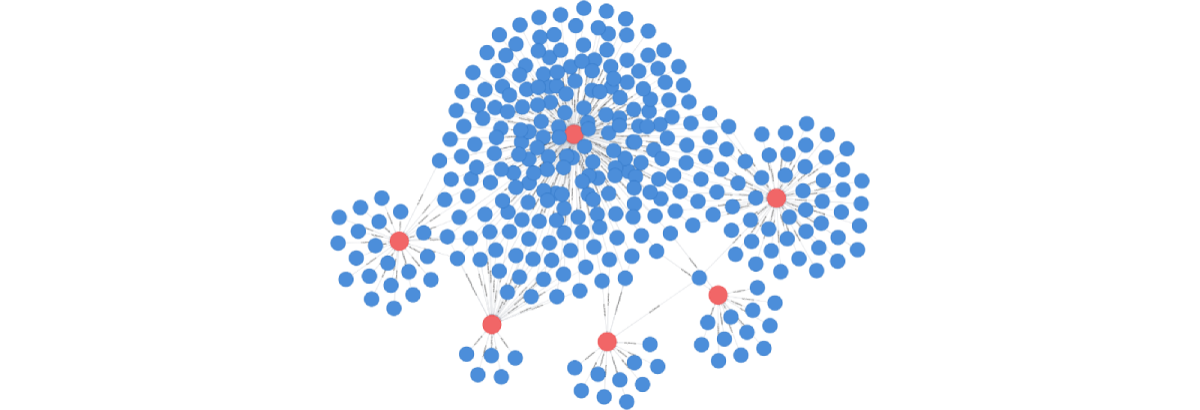
Topology of an example patient graph searched with 6 disease factors. Search query 1 in Table 3 was used. Patient nodes are shown in blue and factor nodes are shown in red. Lines represent relationships between a patient and factors.

**Figure 3:**
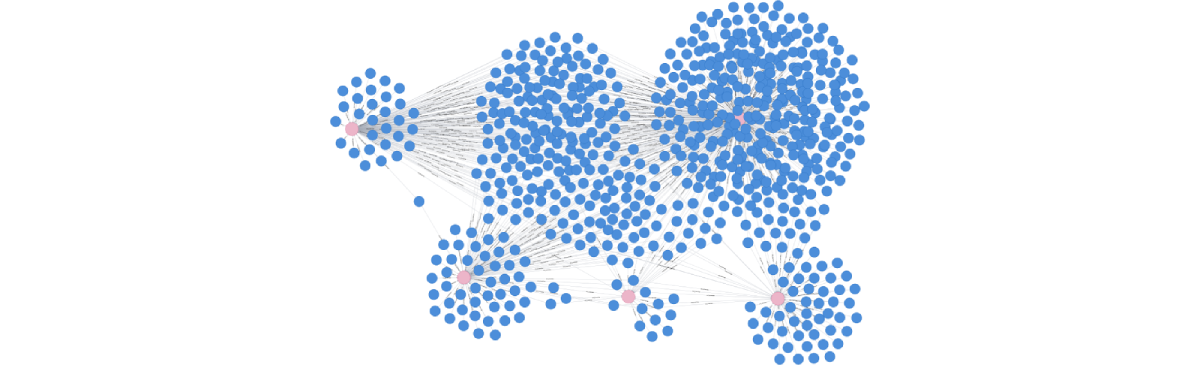
Topology of an example patient graph searched with 5 nonlaboratory factors. Search query 2 in Table 3 was used. Patient nodes are shown in blue and factor nodes are shown in pink. Lines represent relationships between a patient and factors.

**Figure 4:**
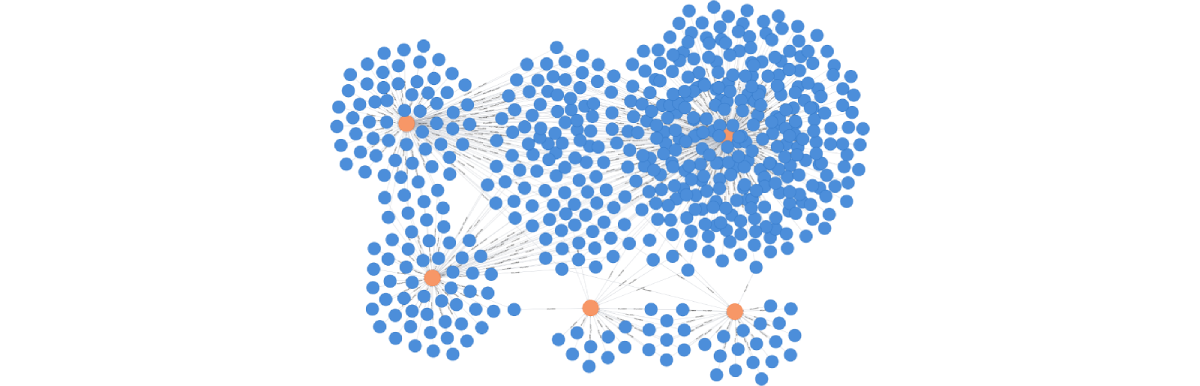
Topology of an example patient graph searched with 5 laboratory factors. Search query 3 in Table 3 was used. Patient nodes are shown in blue and factor nodes are shown in orange. Lines represent relationships between a patient and factors.

The patient health factor graph was constructed by importing patient properties for the patient nodes and factor properties for the corresponding health factor nodes. The resulting patient graph had 93,000 relations between the 2794 patient nodes and 650 factor-value pair nodes. Table 3 lists several examples of Cypher queries for searching patients with various factors. For example, clinicians can easily search for patients with LC with 1 or more co-occurring diseases (Figure 2), with 1 or more nonlaboratory factors (symptoms, medical histories, and observations; Figure 3), or laboratory tests (Figure 4). One can also easily search for any number of health factors shared by patients among patients with LC.

**Table 3:** Examples of graph search tasks and queries using Cypher language.

Number	Graph search task	Cypher query^a,b^
1	Search for patients with LC with 1-6 co-occurring diseases and present the topology. C-389764: Hypocalcemia C-172569: Bacterial Infection C-765209: Obstructive pneumonia C-305976: Pneumothorax C-352894: Leukopenia C-654730: Pneumonia	match (p:Patient {label:'1'})-->(f {cat: 'dac'}) where f.code = 'C-389764' or f.code = 'C-172569' or f.code = 'C-765209' or f.code = 'C-305976' or f.code = 'C-352894' or f.code = 'C-654730' return p, f;
2	Search for patients with LC with 1-5 nonlaboratory factors and present the topology C-549780: Pain C-289547: Bloodstained sputum C-127089: Hoarseness C-029761: Productive Cough C-294680: Swollen Lymph Node in head and neck	match (p:Patient {label:'1'})-->(f) where (f.code = 'C-549780' and f.valcvt = 'true') or (f.code = 'C-289547' and f.valcvt='true') or (f.code = 'C-127089' and f.valcvt='true') or (f.code = 'C-029761' and f.valcvt='true') or (f.code = 'C-294680' and f.valcvt='true') return p, f;
3	Search for patients with LC with 1-5 laboratory test values and present the topology. C-659218: Hepatitis B virus C-493765: Squamous cell carcinoma antigen C-573086: Neuron-specific enolase measurement C-120948: Gastrin-releasing peptide precursor increased C-814793: Mycoplasma pneumoniae antibody	match (p:Patient {label:'1'})-->(f {cat: 'lab'}) where (f.code = 'C-659218' and f.valcvt = 'true') or (f.code = 'C-493765' and f.valcvt = 'up') or (f.code = 'C-573086' and f.valcvt = 'up') or (f.code = 'C-120948' and f.valcvt = 'abnormal') or (f.code = 'C-814793' and f.valcvt = 'abnormal') return p, f;
4	Search for 1 patient, show the electronic medical record health factor graph and the Unified Medical Language System knowledge graph together	match (p:Patient {label:'1', vpid:'_8908085766'})-->(f) match (p)-->(ap:AbstractPatient)-->(tc:TargetConcept)-->(cr:RelCat)-->(c:Concept) return p, f, ap, tc, cr, c;

^a^Using Neo4j Cypher query language.

^b^Patient with LC: label=1; background patient: label=0. Factor property f.code: unique local code. Factor property f.valcvt: converted value.

### Integration of the EMR Patient Graph With the UMLS Subgraph

**Figure 5:**
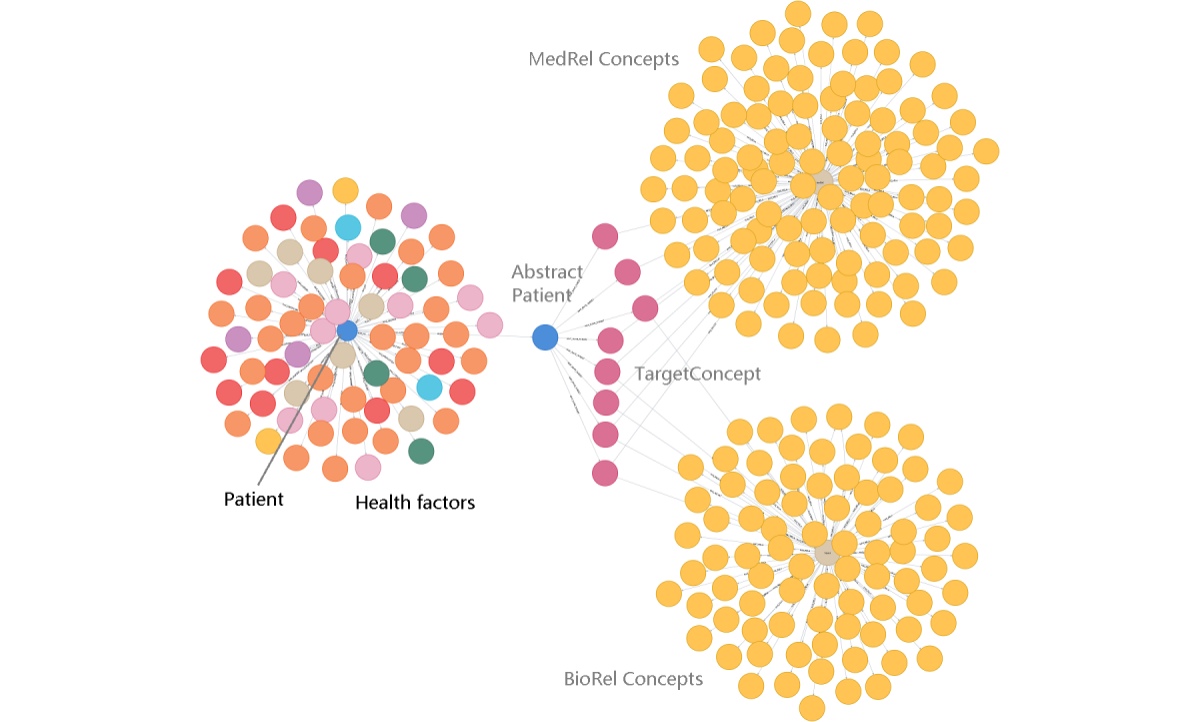
Example search result of the integrated biomedical graph. Search query 4 in Table 3 was used to search 1 specific ID of a patient with lung cancer. Left side: health factors from the electronic medical record of one patient with lung cancer. Right side: lung cancer biomedical knowledge from the Unified Medical Language System. Middle: single AbstractPatient as the connection. BioRel: biological concept relationship; MedRel: medical concept relationship.

As the largest integrated biomedical ontology, the UMLS graph contains hierarchies of diseases and horizontal relationships with other entities. Within a disease family such as LC, the various types of LCs are horizontally connected to a myriad of related biomedical concepts including genes, proteins, symptoms, observations, medication, and treatments. This study is focused on the UMLS knowledge subgraph containing horizontal relationships for LC. Using the UMLS LC hierarchy, the target LC codes found in EMRs were expanded to 8 main LC concepts (Table 1). From these concepts, approximately 1100 relations were identified in the UMLS ontology. Most of the relations were hierarchical—for example, a parent-child relationship—and thus the relations were further filtered by the biomedical relationships that we were interested in (Textbox 1). The resulting UMLS LC biomedical subgraph had 8 LC concept nodes, 187 related biomedical concepts, and 188 horizontal biomedical relations (Figure 5).

Through a single AbstractPatient node, the EMR patient graph was connected to the UMLS subgraph for LC. Search query 4 in Table 3 and its search result in Figure 5 show an example presentation of both actual patient’s health factors in the EMR and relevant biomedical knowledge in the UMLS in the same graph.

### Generation of the Distribution of LC Health Factors From the EMR

**Figure 6:**
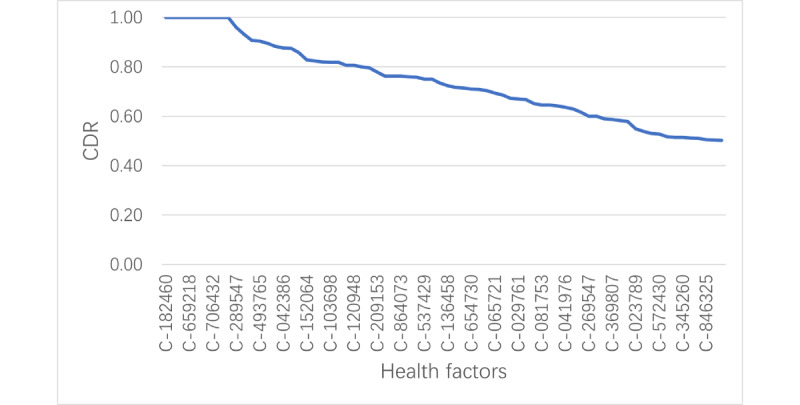
Distribution curve of lung cancer health factors sorted by the connection delta ratio (CDR; cutoff=0.5). Only partial codes are visible on the x-axis. The full spectrum of lung cancer health factors can be found in Table A1 in Multimedia Appendix 1.

With the patient health factor graph, we searched for patients with LC and background patients with each of the health factors and its value. The connection delta ratios were calculated for each factor from the number of connections to patients with LC and the number of connections to background patients. Sorting factors by CDR in descending order generated a distribution of health factors for LC found in the EMR. The complete distribution of top-ranked factors over a CDR cutoff of 0.5 are shown in Table A1 in Multimedia Appendix 1 and plotted in Figure 6. As examples, up to 5 top health factors in each category are shown in Table 4. For understanding LC risk factors, this distribution excluded the various cancers, all procedures and medications related to cancers, and treatments.

**Table 4:** Partial distribution of lung cancer health factors sorted by category and connection delta ratio (cutoff=0.5) as examples. The full distribution of lung cancer health factors is provided in Table A1 in Multimedia Appendix 1.

Category^a^	Local code	Term	Value	Connection delta ratio	Tag
dac	C-182460	Left lung pulmonary obstructive pneumonia	TRUE	1.00	confirmed
dac	C-248056	Right lung pulmonary obstructive pneumonia	TRUE	1.00	confirmed
dac	C-765209	Obstructive pneumonia	TRUE	1.00	confirmed
dac	C-305976	Pneumothorax	TRUE	0.93	correlated
dac	C-172569	Bacterial Infection	TRUE	0.88	correlated
lab	C-659218	Hepatitis B virus	TRUE	1.00	correlated
lab	C-493765	Squamous cell carcinoma antigen	up	0.90	confirmed
lab	C-573086	Neuron-specific enolase measurement	up	0.82	correlated
lab	C-952408	Non-small cell lung cancer associated-antigen	up	0.82	confirmed
lab	C-103698	Superoxide dismutase measurement	down	0.82	correlated
obs	C-039824	Mediastinal mass	TRUE	1.00	confirmed
obs	C-706432	Lung mass	TRUE	1.00	confirmed
obs	C-748932	Lung mass found in checkup	TRUE	1.00	confirmed
obs	C-134276	Lung shadow	TRUE	0.91	confirmed
obs	C-706281	Bronchial stenosis	TRUE	0.89	correlated
rf	C-902187	Smoking	TRUE	0.50	confirmed
smp	C-549780	Pain	TRUE	1.00	confirmed
smp	C-289547	Bloodstained sputum	TRUE	0.96	confirmed
smp	C-152064	Hemoptysis (cough up blood)	TRUE	0.83	correlated
smp	C-243071	Shoulder Pain	TRUE	0.82	confirmed
smp	C-127089	Hoarseness	TRUE	0.80	correlated

^a^Categories include condition (dac), laboratory test (lab), observation (obs), risk factor (rf), and symptom (smp).

We checked the medical literature for any associations between these top CDR-ranked health factors and LC [14-26]. This literature review confirmed that 70 out of the 71 factors (Table A1 in Multimedia Appendix 1) were LC risk factors or were correlated with LC. The relationship between 1 factor, laboratory test for immunoglobulin E levels, and LC was unsure according to the literature [27]. This high degree of concordance between the results of our CDR analysis and the literature suggests that the patient graph CDR method was effective in generating a reliable distribution of LC health factors from EMR patient data.

## Discussion

Using hospital EMR patient data and applying the new patient graph CDR method recently developed from synthetic patient data, this study was able to construct an integrated biomedical graph for LC. From searching the graph, the study created a distribution of health factors for LC, which were verified through literature review. Our results show that the new strategy of first using synthetic patients for method development and then applying the methods with real patient data is valid and effective.

This study has implications for hospitals with regard to harnessing KG databases and technologies. First, generating an integrated biomedical graph with hospital EMR data may enable medical professionals to view individual patient’s health factor graphs along with the related UMLS KGs for comprehensive comparisons. Current medical concept nodes horizontally related to the LC nodes are mostly genes and gene-related biological information, as well as drugs and treatment-related information from the UMLS ontology (see Figure 5). Since the UMLS is updated quarterly, the LC integrated biomedical graph will grow as the UMLS grows. Thus, this KG integration offers a new way for hospitals to bring continuously updated international standard biomedical knowledge to patient care. The current graph model is designed specifically for searching risk factors; however, it can be modified for other clinical information tasks. It may also be integrated with cancer-associated lifestyle KGs for disease management information [28].

The second implication of this study may be applying the CDR-ranked distribution of health factors to build more effective or practical machine learning models for LC risk prediction. Because the distribution ranks factors from higher to lower relative strength, they may be used to help select more health factors to build prediction models; that is, feature engineering. For example, we have an ongoing project experimenting with the factor distribution in building LC risk prediction machine learning models. Knowing the risk factors actually found in the EMR data, we could focus on these risk factors and reduce the variables from over 100 to less than 30 in the machine learning models that were generated from EMR-wide data. To increase the LC screening rate in larger populations, machine learning models with a small number of variables for which data can be readily available in community and rural clinics are necessary.

In addition, the patient health factor graphs generated from EMR data may enable hospitals to study the effect of various types of factors in diagnosis, medication, treatment, and disease management. Such graph analysis complements existing statistical analysis. Traditionally, studies on individual risk factors are hypothesis driven and use a clinical trial or case-control study design [29]. The literature found in this study for verification of the health factor distribution collectively indicate the use of this approach [14-27]. Because this study’s patient graph method is EMR data driven, it can reveal potential new risk factors or inconclusive risk factors that deserve additional research. For example, the factor “laboratory test for immunoglobulin E levels” was tagged as “unsure” in the distribution because prior studies were inclusive. Our CDR analysis suggests that this immunoglobulin E factor requires further clinical validation [30].

Because EMR data sometimes have biases and missing data, the EMR data–driven patient graph CDR method has limitations. CDR is a simple measurement of a factor’s relative strength, but caution should be taken when considering factors with a high CDR but a small number of connections. The higher the number of connections, the more reliable the CDR. Hence, studies should set a cutoff for the CDR as well as the minimal number of connections to ensure that the study uses enough data. It is also important to recognize factors that might be affected by data biases and to exclude them from CDR analysis [31]. For EMRs lacking standardized and structured data, collecting standardized data is crucial but challenging. If a data collection pipeline is not fully automated, collecting enough unbiased standardized patient profile data will be a very time-consuming process.

In conclusion, by collecting standardized data of thousands of patients with and those without LC from EMRs, it was possible to generate a hospital-wide patient-centered health factor graph for graph search and presentation. It was also practical to integrate the patient graph with the UMLS KG for LC, enabling hospitals to bring continuously updated international standard biomedical KGs from the UMLS to clinical care. Applying CDR analysis to the graph of patients with LC yielded a CDR-sorted distribution of health factors, where top CDR-ranked health factors showed a high degree of concordance with the literature. The resulting distribution of LC health factors can be used to help personalize risk evaluation and preventive screening recommendations.

## Appendix

Multimedia Appendix 1 Complete lung cancer health factor distribution sorted by category (Cat) and connection delta ratio (CDR).

## Abbreviations

CDR: connection delta ratio
EMR: electronic medical record
KG: knowledge graph
LC: lung cancer
PDJ: patient diagnosis journey
UMLS: Unified Medical Language System
