# Supplementary material for: The Generation of a Lung Cancer Health Factor Distribution Using Patient Graphs Constructed From Electronic Medical Records: Retrospective Study
Source: J Med Internet Res. 2022 Nov 25;24(11):e40361. doi: 10.2196/40361 (PMC9736747; doi:10.2196/40361)
Supplement: Multimedia Appendix 1 [file jmir_v24i11e40361_app1.pdf]

Appendix

Table A1
Complete lung cancer health factor distribution sorted by category (Cat) and connection delta ratio (CDR).

Cat	Term	Value	Patient Count	Control Count	CDR	Tag
dac	Left lung pulmonary obstructive pneumonia	TRUE	81	0	1.00	confirmed
dac	Right lung pulmonary obstructive pneumonia	TRUE	104	0	1.00	confirmed
dac	Obstructive pneumonia	TRUE	59	0	1.00	confirmed
dac	Pneumothorax	TRUE	28	1	0.93	correlated
dac	Bacterial Infection	TRUE	15	1	0.88	correlated
dac	Hypocalcemia	TRUE	28	3	0.81	correlated
dac	Pulmonary tuberculosis	TRUE	37	5	0.76	confirmed
dac	Community acquired pneumonia	TRUE	103	17	0.72	correlated
dac	Pneumonia	TRUE	212	36	0.71	confirmed
dac	Hypoalbuminemia	TRUE	59	11	0.69	correlated
dac	Ventilatory defect	positive	97	22	0.63	correlated
dac	Bulla of lung	TRUE	59	14	0.62	correlated
dac	Arteriosclerosis	TRUE	12	3	0.60	correlated
dac	Bronchitis	TRUE	49	15	0.53	confirmed
dac	Pulmonary emphysema	TRUE	304	94	0.53	confirmed
dac	Chronic bronchitis	TRUE	22	7	0.52	confirmed
dac	Hypokalemia	TRUE	25	8	0.52	correlated
dac	Right lung pneumonia	TRUE	75	24	0.52	confirmed
dac	Pharyngitis	TRUE	34	11	0.51	correlated
dac	Infectious disease of lung	TRUE	127	42	0.50	confirmed
lab	Hepatitis B virus	TRUE	26	0	1.00	correlated
lab	Squamous cell carcinoma antigen	up	139	7	0.90	confirmed
lab	Neuron-specific enolase measurement	up	394	38	0.82	correlated
lab	Non-small cell lung cancer associated-antigen	up	578	57	0.82	confirmed
lab	Superoxide dismutase measurement	down	20	2	0.82	correlated
lab	Gastrin-releasing peptide precursor increased	up	168	18	0.81	confirmed

lab	Mycoplasma pneumoniae antibody 1:40 dilution	abnormal	89	12	0.76	correlated
lab	Mycoplasma pneumoniae antibody (passive agglutination)	abnormal	89	12	0.76	correlated
lab	Mycoplasma pneumoniae AbIgM	abnormal	44	6	0.76	correlated
lab	Cancer antigen 15-3 measurement	up	247	34	0.76	confirmed
lab	Mycoplasma pneumoniae antibody 1:80 dilution	abnormal	50	8	0.72	correlated
lab	Alpha-2 antiplasmin functional assay	up	72	13	0.69	correlated
lab	Squamous cell carcinoma antigen level	down	92	18	0.67	confirmed
lab	Tuberculosis T-cell test	abnormal	79	17	0.65	confirmed
lab	Immunoglobulin E measurement	up	58	15	0.59	unsure
lab	Human epididymis protein 4	up	50	13	0.59	confirmed
lab	Mycoplasma pneumoniae antibody 1:160 dilution	abnormal	19	5	0.58	confirmed
lab	Antithrombin III assay	up	45	12	0.58	correlated
lab	Carcinoembryonic antigen measurement	up	567	165	0.55	confirmed
lab	Mycoplasma pneumoniae antibody 1:320 dilution	abnormal	10	3	0.54	correlated
lab	Platelet-large cell ratio decreased	down	67	22	0.51	correlated
obs	Mediastinal mass	TRUE	25	0	1.00	confirmed
obs	Lung mass	TRUE	45	0	1.00	confirmed
obs	Lung mass found in checkup	TRUE	76	0	1.00	confirmed
obs	Lung shadow	TRUE	41	2	0.91	confirmed
obs	Bronchial stenosis	TRUE	18	1	0.89	correlated
obs	Lung nodules	TRUE	466	29	0.88	confirmed
obs	Enlarged lymph nodes inside chest	TRUE	212	14	0.88	confirmed
obs	Enlarged adrenal gland	TRUE	26	2	0.86	correlated
obs	Atelectasis	TRUE	158	18	0.80	correlated
obs	Adrenal mass	TRUE	16	2	0.78	correlated
obs	Adrenal nodule	TRUE	21	3	0.75	correlated
obs	Pericardial effusion	TRUE	111	17	0.73	correlated
obs	Enlargement of lymph nodes	TRUE	60	10	0.71	confirmed
obs	Left lung atelectasis	TRUE	23	4	0.70	correlated
obs	Pleural effusion	TRUE	332	70	0.65	correlated
obs	Right lung atelectasis	TRUE	51	11	0.65	correlated
obs	Cerebral ischemia	TRUE	31	10	0.51	correlated
rf	Smoking	TRUE	282	93	0.50	confirmed

smp	Pain	TRUE	77	0	1.00	confirmed
smp	Bloodstained sputum	TRUE	192	4	0.96	confirmed
smp	Hemoptysis (cough up blood)	TRUE	106	10	0.83	correlated
smp	Shoulder Pain	TRUE	10	1	0.82	confirmed
smp	Hoarseness	TRUE	27	3	0.80	correlated
smp	Purulent sputum	TRUE	14	2	0.75	confirmed
smp	Back Pain	TRUE	41	7	0.71	confirmed
smp	Productive Cough	TRUE	541	107	0.67	confirmed
smp	Swollen Lymph Node in head and neck	TRUE	130	26	0.67	confirmed
smp	Cough	TRUE	733	160	0.64	confirmed
smp	Productive cough -clear sputum	TRUE	252	56	0.64	confirmed
smp	Night sweats	TRUE	16	4	0.60	confirmed

Cat: Categories, including condition (dac), lab test (lab), observation (obs), risk factor (rf), symptom (smp).

Patient count: Number of lung cancer patients connected to a factor.

Control count: Number of control patients connected to a factor.

CDR: Connection delta ratio, cutoff = 0.5.
